# Intervertebral disc disease in Dachshunds radiographically screened for intervertebral disc calcifications

**DOI:** 10.1186/s13028-014-0089-4

**Published:** 2014-12-19

**Authors:** Anu K Lappalainen, Elina Vaittinen, Jouni Junnila, Outi Laitinen-Vapaavuori

**Affiliations:** Section of Small Animal Surgery, Department of Equine and Small Animal Medicine, Faculty of Veterinary Medicine, University of Helsinki, P.O. Box 57, FI - 00014 HU Helsinki, Finland; Heinolan Eläinlääkäriasema, Reumantie 2, FI - 18100 Heinola, Finland; Pharma Ltd., Espoo, Finland

**Keywords:** Intervertebral disc calcification, Intervertebral disc disease, Canine, Radiographic screening

## Abstract

**Background:**

Intervertebral disc disease (IDD) is a very common neurological disease, Dachshunds being the breed most often affected. In this breed, IDD has a hereditary background and is associated with intervertebral disc calcification (IDC), an indicator of severe intervertebral disc degeneration. In Finland, spinal radiography is used, when screening for IDC before breeding Dachshunds. We evaluated the association between IDC and IDD in Finnish Dachshunds radiographically screened for IDC.

A questionnaire was sent to owners of 193 radiographically screened Dachshunds aged at least ten years. Clinical signs indicative of IDD were compared with IDC grade (grade 0 = no calcifications, grade 1 = 1 – 2 calcifications, grade 2 = 3 – 4 calcifications and grade 3 = 5 or more calcifications) and with age at the time of the radiographic examination. The diagnosis of IDD was confirmed by a veterinarian.

**Results:**

IDD was common in the study population with 31% of dogs being affected. IDD and IDC were clearly connected (*P* < 0.001); IDD was rare in dogs with no calcifications (grade 0) and common in dogs with severe IDC (grade 3). The IDC grade was strongly positively associated with frequency of back pain periods (*P* < 0.001), and dogs with IDC grade 3 had frequent periods of pain. Reluctance to jump onto a sofa had a strong positive association with back pain. No association existed between age of the dog at the time of the radiographic examination and clinical signs indicative of IDD.

**Conclusions:**

Radiographically detected IDC and IDD are common in Finnish Dachshunds and are strongly associated with one another. Spinal radiography is an appropriate screening tool for breeders attempting to diminish IDC and IDD in Dachshunds. A breeding program that screens dogs and selects against IDC can be expected to reduce the occurrence of IDD in future. Twenty-four to 48 months of age is a suitable age for screening.

**Electronic supplementary material:**

The online version of this article (doi:10.1186/s13028-014-0089-4) contains supplementary material, which is available to authorized users.

## Background

Intervertebral disc disease (IDD), often causing devastating back pain and neurological deficits such as paresis or paralysis, is a major medical condition in Dachshunds [1]. The peak incidence of the disease has been reported to vary between four and six years [2,3]. Studies of prevalence of IDD are scarce, but estimates have ranged from 19% to 36% [1,4-7]. In a study based on Swedish insurance data, miniature Dachshunds had the highest mortality rate of IDD of all breeds [4].

Intervertebral disc calcification (IDC) is regarded as part of the disc degeneration process in both man and dog [8-12]. In children, it is a rare condition of unknown origin, occurring most often in the cervical spine and causing severe pain [13-15]. The calcifications usually disappear [14], but they can remain visible for several years [15]. Dachshunds are predisposed to early intervertebral disc degeneration and calcification, and IDC can sometimes be seen macroscopically already at the age of nine months [12]. In Dachshunds, most radiographically visible disc calcifications can be seen by two years of age [16]. A disc calcification can disappear after herniation, but resorption of the most degenerated discs is also possible. This has been postulated to be caused by an inflammatory response and phagocytic resorption of the calcified material in the nucleus after tearing of the degenerated annulus fibrosus [17]. Several studies have demonstrated a familial background for IDD and IDC in Dachshunds [18-23]. The hereditary basis of IDC was shown in a recent genetic study in this breed in which a major locus on chromosome 12 was found to harbour genetic variations that affected the development of IDC [24,25].

An association between the existence or number of calcifications and risk for IDD has been previously shown; in a population of Danish Dachshunds, the lower the number of calcifications, the smaller the risk for IDD [5]. In a study of Finnish miniature Dachshunds, only one out of 25 dogs without calcifications had had signs of IDD [26]. The association between IDC and IDD has been established as a tool to reduce the occurrence of IDD. Radiographic screening for IDC has been recommended in three Nordic countries (Denmark, Finland and Norway). In Finland, the protocol includes laterolateral radiographs of the cervical, thoracic and lumbar spine. IDC is graded as follows: no calcifications = free (IDC 0), 1 – 2 calcifications = mild (IDC 1), 3 – 4 calcifications = moderate (IDC 2) and ≥ 5 calcifications = severe (IDC 3). The same grading is used also in Denmark and Norway. The preferred age range for screening in Finland is 24 – 42 months, but Dachshunds aged between 12 and 24 months old or older than 42 months have also been radiographed. In Denmark and Norway, the preferred age range is set at 24 – 48 months.

In Finland, a radiographic screening scheme for IDC in Dachshunds has been in use for over a decade. The aim of our study was to inspect the association between IDC and IDD in Finnish Dachshunds radiographically screened for IDC during a ten-year follow-up period. The hypothesis was that IDD is more common in dogs with severe IDC than in dogs only mildly affected or unaffected. The effect of age of the dog at the time of the radiographic examination on IDC and IDD were also studied.

## Methods

Dachshunds aged at least ten years and screened for IDC according to the Finnish Dachshund Club’s scheme at the age of at least 12 months were included in the study. A multiple-choice questionnaire (Additional file 1), similar to one used in a previous study of incidence of IDC in Finnish miniature Dachshunds [26], was applied to determine the occurrence of IDD in Dachshunds. The questionnaire comprised the owners’ assessment of any clinical signs of IDD (ataxia of hind limbs, back or neck pain, unexplained pain attacks, unwillingness to jump onto a sofa), and their frequency (never, seldom, sometimes, often, always) in their dogs. The owners were also asked about the age at clinical sign onset and whether the dog had been diagnosed by a veterinarian and treated for the clinical signs. The dog was recorded as positive for IDD if it had had neurological deficits of the limbs or pain focusing on the back or neck, and the diagnosis had been confirmed by a veterinarian by clinical examination or in surgery. Frequency of the clinical signs indicating IDD were scored (never = 0, seldom = 1, sometimes = 2, often = 3, always = 4) and answers to questions 1 – 3 (back or neck pain, unexplained pain attacks, unwillingness to jump onto a sofa) in the questionnaire were summed up to get an overall score for symptoms indicating pain.

Altogether 386 Dachshunds met the inclusion criteria and the questionnaire was sent to their owners. Of these owners, 213 (55%) returned the questionnaire. Twelve dogs had died before ten years of age for reasons unrelated to spinal diseases, and they were excluded from the study, as were two dogs with unclear information given by their owners. Six dogs had signs indicative of IDD (pain of the back or neck, ataxia of hind limbs) without a diagnosis by a veterinarian, and they were excluded too. Thus, 193 dogs were included in our study. The IDC grade, number and the age when the radiographic screening was performed were received from the Finnish Dachshund Club open database.

The dogs were divided into three groups based on age when the radiographs were taken. This was done to explore whether age at the time of radiographic examination had an effect on the association between IDC and owner-assessed signs indicative of IDD. In Group 1, the dogs were radiographed at the age of 12 – 24 months, in Group 2 at 24 – 48 months and in Group 3 at more than 48 months.

All statistical analyses were conducted using SAS® System for Windows, version 9.3 (SAS Institute Inc., Cary, NC, USA). The association between IDC grade/number was analysed with a logistic regression model. IDC grade/number was used as the sole fixed effect in this model. The effect of age on the IDC grade and the effect of IDC grade on the frequency of clinical signs indicating IDD were assessed with cumulative logit models. In the latter, for analysis purposes, the frequency classes with very low number of observations were combined (“sometimes” and “often” in the back or neck pain and ataxia of hind limbs, and “often” and “always” in reluctance to jump onto a sofa). The cumulative logit models included the IDC grade and the age group (Groups 1 – 3) as fixed effects. If the effect of age group was statistically insignificant, a similar cumulative logit model was fitted with only the IDC grade as a fixed effect. The association between reluctance to jump onto a sofa and the back or neck pain was assessed with Fisher’s exact test.

In the logistic regression and cumulative logit models, the differences between groups were quantified with odds ratios (ORs) and their 95% confidence intervals (CIs). P-values ≤ 0.05 were considered statistically significant. All models were constructed to model the probabilities of higher/worse values in response, and the ORs were derived accordingly.

## Results

The most common breed variant amongst the 193 Dachshunds was standard wire haired Dachshund (n = 63) followed by miniature long haired Dachshund (n = 38). The breed variant distribution of the dogs is presented in Table 1. The median age at the radiographic screening was 50 (range 12 – 116) months. Thirty dogs (15.5%) were radiographed at the age of 12 – 24 months (Group 1), 114 (59.1%) at 24–48 months (Group 2) and 49 (25.4%) at more than 48 months (Group 3). Forty-four dogs (22.8%) were classified as grade 0 (free of calcifications), 81 (42.0%) as grade 1 (1 – 2 calcifications), 29 (15.0%) as grade 2 (3 – 4 calcifications) and 39 (20.2%) as grade 3 (≥5 calcifications). IDC grade and age group were associated (p = 0.001), as more calcifications were detected in 24 – 48 months old dogs compared to the other two age groups. The detailed information of distribution of IDC grades according to age group is presented in Table 2. The median number of calcifications per dog was 1.0 (range 0 – 11) in Group 1, 2.0 (range 0 – 12) in Group 2 and 1.0 (range 0 – 13) in Group 3.

**Table 1:** **Breed variant distribution and status of intervertebral disc disease in 193 Dachshunds radiographed for intervertebral disc calcifications**

**Breed variant**	**Dogs**	**IDD**	**Free of IDD**	**IDD%**
	**n**	**n**	**n**	
Standard wire-haired	63	18	45	28.6
Miniature wire-haired	30	15	15	50.0
Standard smooth-haired	26	7	19	26.9
Miniature smooth-haired	6	2	4	33.3
Standard long-haired	30	4	26	13.3
Miniature long-haired	38	13	25	34.2
**Total number**	**193**	**59 (31%)**	**134 (69%)**	

n = number of dogs, IDD = intervertebral disc disease, IDD% = proportion of dogs with IDD.

**Table 2:** **Number of dogs with intervertebral disc calcification grades 0 – 3; Grade 0 (0 calcifications), Grade 1 (1 – 2 calcifications), Grade 2 (3 – 4 calcifications), Grade 3 (≥5 calcifications) in the three age groups in 193 Dachshunds radiographed for intervertebral disc calcifications**

**Age (months)**	**Grade 0**	**Grade 1**	**Grade 2**	**Grade 3**	**Total**
	**(0 calc)**	**(1–2 calc)**	**(3–4 calc)**	**(≥5 calc)**	
	**n (%)**	**n (%)**	**n (%)**	**n (%)**	
12 – 24	14 (47)	10 (33)	3 (10)	3 (10)	30 (15.5)
24 – 48	16 (14)	49 (43)	22 (19)	27 (24)	114 (59.1)
>48	14 (29)	22 (45)	4 (8)	9 (18)	49 (25.4)
**Total**	**44 (22.7)**	**81 (42.0)**	**29 (15.0)**	**39 (20.2)**	**193 (100)**

n = number of dogs, calc = calcification.

**Figure 1:**
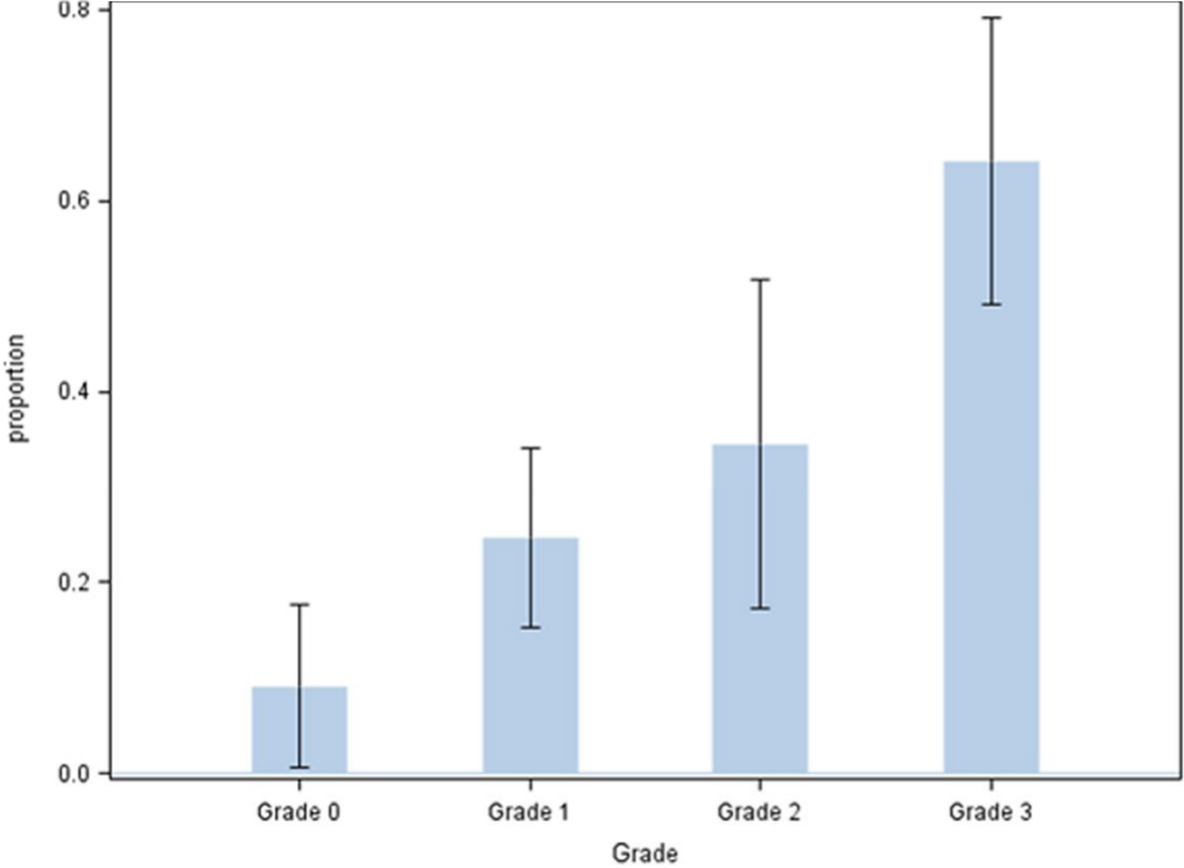
**Proportion of dogs with intervertebral disc disease (IDD) in intervertebral disc calcification (IDC) Grade 0 (0 calcifications), Grade 1 (1 – 2 calcifications), Grade 2 (3 – 4 calcifications), Grade 3 (≥5 calcifications) of the 193 Dachshunds radiographed for intervertebral disc calcification.** Whiskers = CI 95%.

Of the 193 dogs, 59 (31%) had an IDD diagnosis made by a veterinarian (Table 1). Forty-three dogs (73%) had been treated conservatively with rest and non-steroidal anti-inflammatory drugs, 12 (20%) surgically and 4 (7%) had been euthanized because of the disease. One dog was operated twice and then euthanized because of the IDD, and is counted only in the surgically treated group. The number and proportion of dogs with and without IDD in IDC grades 0 – 3 are presented in Figure 1.

According to the owners, the most common clinical signs of IDD were the back or neck pain (52 dogs, 88%) and unwillingness to jump onto a sofa (48 dogs, 81%). Altogether 37 dogs (63%) with IDD diagnosed by a veterinarian had had at least one episode of some degree of neurological deficits of the hind limbs; the rest had suffered from back pain only. Based on the information given by 28 owners, the median age for IDD was 72 months (range 30 – 132). The mean of summed score for answers to questions 1 – 3 in the questionnaire was 2.9 (range 0 – 7) for the dogs with IDD and 0.4 (range 0 – 6) for the dogs free of IDD. In the latter group the dog with the highest score had been diagnosed having a recurrent otitis media, and the clinical signs were due to that disease. Of the 134 dogs free of IDD, 104 (78%) had a sum score of 0. That is, owners had answered “never” to all three questions.

The IDC grade was positively associated (*P* < 0.001) with IDD in 193 Dachshunds (Table 3). Dogs with severe IDC (grade 3) had 17.9-fold odds for IDD compared with dogs without calcifications (grade 0). Only grades 1 and 2 did not differ statistically from each other. Of the 16 surgically treated or euthanized dogs, eight (50%) were graded as IDC 3, three (19%) as IDC 2 and five (31%) as IDC 1. Also the number of IDC was positively associated with IDD (*P* < 0.001) with OR of 1.3 (1.2 – 1.5) per calcified disc. IDC grade significantly explained three of the four clinical signs indicative of IDD (reluctance to jump onto a sofa, back or neck pain, pain attack for unknown reason) (Table 4). The effects were strongest between grade 3 and grade 0. For example, the proportion of dogs suffering from back pain more frequently was ten-fold when the proportion of dogs with IDC grade 3 were compared with the proportion of dogs with IDC Grade 0 (*P* < 0.001). The overall effect of IDC grade on ataxia of hind limbs was only slightly above significance (*P* = 0.061).

**Table 3:** **Effect of intervertebral disc calcification (IDC) grade on intervertebral disc disease (IDD) in 193 Dachshunds radiographed for IDC**

**Grading of calcification (IDC grade)**	**OR (CI 95%)**
1 versus 0	3.3 (1.0 – 1.4)*
2 versus 0	5.3 (1.5 – 19.1)**
2 versus 1	1.6 (0.6 – 4.0)ns
3 versus 0	17.9 (5.2 – 60.9)**
3 versus 1	5.4 (5.2 – 12.5)**
3 versus 2	3.4 (1.2 – 9.4)*

Disease was measured by a questionnaire where the owner was asked to report symptoms indicative of IDD and also confirmed and treated by veterinarians. Grade 0 (0 calcifications), Grade 1 (1 – 2 calcifications), Grade 2 (3 – 4 calcifications), Grade 3 (≥5 calcifications).

OR = odds ratio, CI = confidence interval, * = *P* ≤ 0.05, ** = *P* ≤ 0.01, ns = not significant.

**Table 4:** **Effect of intervertebral disc calcification (IDC) grade on the frequency of back or neck pain, frequency of pain attacks of unknown origin, frequency of ataxia of hind limbs and frequency of unwillingness to jump onto a sofa in 193 Dachshunds radiographed for IDC**

**Grading of calcification (IDC grade)**	**OR (CI 95%) back or neck pain**	**OR (CI 95%) pain attacks of unknown origin**	**OR (CI 95%) ataxia of hind limbs**	**OR (CI 95%) unwillingness to jump onto a sofa**
1 versus 0	2.9 (1.0 – 8.4)*	3.3 (1.0 – 10.4)*	1.2 (0.4–3.1)ns	2.1 (0.9 – 5.3)ns
2 versus 0	4.3 (1.3 –14.5)*	3.2 (0.83 – 12.2)ns	1.06 (0.3 – 3.8)ns	1.9 (0.6 – 5.6)ns
2 versus 1	1.5 (0.6 – 3.7)ns	1.0 (0.4 – 2.7)ns	0.9 (0.3 – 2.8)ns	0.9 (0.4 – 2.2)ns
3 versus 0	10.1 (3.3 – 31.0)**	5.4 (1.6 – 18.5)**	3.8 (1.3 – 10.8)*	5.3 (2.0 – 14.4)**
3 versus 1	3.5 (1.6 – 7.7)**	1.7 (0.7 – 3.8)ns	3.2 (1.4 – 7.7)**	2.5 (1.1 – 5.3)*
3 versus 2	2 2.3 (0.9 – 6.2)ns	1.7 (0.6 – 5.0)ns	3.6 (1.1 – 11.6)*	2.8 (1.0 – 7.7)*

OR = odds ratio, CI = confidence interval, * = *P* ≤ 0.05, ** = *P* ≤ 0.01, ns = not significant.

No statistically significant difference in distribution of severity or frequency of clinical signs indicative of IDD existed between the three age groups. The frequency of back pain was positively associated (*P* < 0.001) with reluctance to jump onto a sofa. In other words, dogs that experienced back pain more frequently were also more often reluctant to jump onto a sofa according to the owners’ evaluation.

## Discussion

The relationship between IDD and radiographically detected IDC was investigated in 193 Finnish Dachshunds screened for IDC. All dogs were older than ten years and had been radiographed for screening purposes according to the Finnish Dachshund Club protocol. The study was conducted as a questionnaire with a response rate of 55%, which can be considered reasonable [27]. Our findings suggest that IDC grade predicts well the probability of manifesting signs indicative of IDD later in life (*P* < 0.001). Only four (9%) of the 44 Dachshunds without IDC (grade 0) had had signs of IDD, whereas 26 (64%) of the 39 dogs classified as grade 3 (≥5 calcifications) had suffered from IDD. Additionally, the dogs with IDC grade 3 had more often and more severe signs of IDD than the dogs with no or only a few calcifications. Our results are in accordance with a previous study conducted on 61 Dachshunds aged at least eight years where the number of radiographically detected IDC, at the age of 24 months was shown to predict well later clinical disease. Also in that study, a clear association between number of calcified discs and IDD existed (*P* < 0.001); in dogs with less than three calcifications, IDD was rare and the clinical signs were less severe [5].

It could be assumed that IDD was more common in the dogs radiographed at the age of 24 – 48 months, as dogs in this group had more calcifications than the dogs in the other two groups. However, severity and frequency of signs indicative of IDD were similarly distributed in all age groups. The reason for this remains unclear.

In this study, 31% (59/193) of the Dachshunds had had signs of IDD. The study was conducted as a questionnaire, and it can be argued that the type of the study is prone to errors of interpretation. The questionnaire has been used in a previous study [25], but it has not been validated, which can be considered a limitation of the study. However, the diagnosis was confirmed by a veterinarian in all cases. It is possible that some of the cases, not confirmed by myelography, CT or MRI, were false positives. However, we did not want to exclude dogs diagnosed without these modalities, since advanced imaging is seldom performed if the dog has mild clinical signs or the owner declines surgery. Only dogs with neurological deficits of the limbs or pain focusing on the back or neck were considered positive for IDD. Some of the dogs with pain of unknown origin or reluctance to jump onto the sofa might have had a mild form of IDD, and were falsely classed as negative for IDD. Also, the owners were aware of the IDC grade of their dogs, and this could have had an influence how they interpret the behavior of their dog, or how the veterinarian interpret the clinical signs.

The incidence of IDD in our study was clearly higher than the typically referred 19% [6]. In a more recent study [5], the occurrence (33%) was similar to that here. However, drawing conclusions on the occurrence of IDD in the general Finnish Dachshund population is not possible since the dogs included in the study might not be a representative sample. In our dog population, IDD appeared to be most common in miniature wire haired Dachshunds (50%) and least common in standard long haired Dachshunds (13%).

In the questionnaire, one of the questions was about a dog’s unwillingness to jump onto a sofa. This question is relevant only if the dog was allowed to jump before the clinical onset of IDD. However. none of the owners marked this question as “always” which would indicate that the dog never does jump onto a sofa. A high positive association existed between the answer to this question and the answer to the question on back pain. It is not clear whether the owners connected unwillingness to jump onto a sofa to back pain, or whether they dealt with these as separate issues. However, the simple question of jumping onto a sofa could be useful as part of the clinical workup of Dachshunds suffering from mild clinical signs of IDD. The question on pain attacks of unknown origin might have been difficult to interpret by some owners. However, it too was associated with IDD, and dogs with several calcifications (grade 3) had them more often than dogs with no or a couple of calcifications (IDC grade 0 and 1). This suggests that dogs with IDC grade 3 might have clinical signs of IDD more often than the owners recognize.

Based on the information from only 28 dogs (14%), the median age when clinical signs of IDD was manifested 72 months (range 30 – 132). The age was higher than that previously reported [2,3], but the result may be inaccurate, since it was based on owners’ recollection. It is possible that the owners remembered best the latest disease episode if there had been several.

Our results support radiography as an effective screening tool for IDC and also by implication as a tool to diminish incidence of IDD in Dachshunds. Our results of a high positive association between IDC and IDD are in accordance with a previous study based on a smaller population [5]. Research on the benefits of radiographic screening as a tool to diminish IDD in Dachshunds has been lacking. However, in a recent study on Danish Dachshunds it has been shown that breeding value based on IDC indicates the risk of offspring’s IDD [7]. CT has been suggested as a screening tool since it is more sensitive than radiography in detecting small calcifications [29]. The most sensitive method for detecting intervertebral disc degeneration is MRI, which also allows degeneration of the disc without mineralization to be seen [30-32]. However, these two methods are expensive and their availability is limited, making them less suitable for screening purposes, where cost and access of diagnostic tools are important issues. Radiography is quite insensitive in detecting small calcifications, as shown in a study comparing radiological and histopathological findings of calcifications. The sensitivity was 60% and specificity 100% for radiography when histopathology was used as the gold standard [28]. Moderate sensitivity of radiography could be considered as a weakness of screening programs, but as it is shown here and in a previous studies [5,29], the number of radiographically visible IDCs is clearly associated with IDD. This indicates that radiography is an adequate modality for screening purposes.

## Conclusions

IDC and IDD are common in Finnish Dachshunds and are strongly associated with one another. Spinal radiography is an appropriate screening tool for breeders attempting to diminish IDC and IDD in Dachshunds. A breeding program that screens dogs and selects against IDC can be expected to reduce the occurrence the occurrence of IDD in future. Twenty-four to 48 months is a suitable age for screening.

## Additional file

Additional file 1:
**A questionnaire for the owners of the ≥ 10 years old Dachshunds screened for intervertebral disc calcifications.**
