# Supplementary material for: Intervertebral disc disease in Dachshunds radiographically screened for intervertebral disc calcifications
Source: Acta Vet Scand. 2014 Dec 19;56(1):89. doi: 10.1186/s13028-014-0089-4 (PMC4285634; doi:10.1186/s13028-014-0089-4)
Supplement: Additional file 1: — A questionnaire for the owners of the ≥ 10 years old Dachshunds screened for intervertebral disc calcifications. [file 13028_2014_89_MOESM1_ESM.docx]

1(1)

**A questionnaire for the owners of the ≥ 10 years old Dachshunds screened for intervertebral disc calcifications**

The dog is alive  dead

The reason for death/euthanasia: ___________________________________________________

*Please answer the following questions if you still own the dog or if you know its disease history. Mark only one answer in the multiple choice questions.*

*.*

**Has your dog had following clinical signs:**

1) Unwillingness to jump onto e.g. a sofa

seldom now and then sometimes often always

2) Back or neck pain

seldom now and then sometimes often always

3) Unexplained pain attacks

seldom now and then sometimes often always

4) Ataxia when walking

seldom now and then sometimes often always

5) Has it been paralyzed? Yes  No

When?__________________________________________________________________________

How did it recover?

________________________________________________________________________________

6) Has it been seen by a veterinarian because of the aforementioned reasons?

Yes  No

What was the diagnosis?____________________________________________________________

What was the treatment? ___________________________________________________________

_______________________________________________________________________________

________________________________________________________________________________
